# Sulfonylurea and Cancer Risk Among Patients With Type 2 Diabetes: A Population-Based Cohort Study

**DOI:** 10.3389/fendo.2022.874344

**Published:** 2022-06-30

**Authors:** Houyu Zhao, Zhike Liu, Lin Zhuo, Peng Shen, Hongbo Lin, Yexiang Sun, Siyan Zhan

**Affiliations:** ^1^ Department of Epidemiology and Biostatistics, School of Public Health, Peking University, Beijing, China; ^2^ Research Center of Clinical Epidemiology, Peking University Third Hospital, Beijing, China; ^3^ Yinzhou District Center for Disease Control and Prevention, Ningbo, China; ^4^ Center for Intelligent Public Health, Institute for Artificial Intelligence, Peking University, Beijing, China

**Keywords:** sulfonylurea, cancer, type 2 diabetes, time-varying confounding, pharmacoepidaemiology

## Abstract

**Background:**

Current evidence of the association between the use of sulfonylurea and cancer risk is highly conflicting and little evidence of this association is from the mainland Chinese population. This study aimed to evaluate the potential effects of sulfonylurea use on cancer risk among patients with type 2 diabetes mellitus (T2DM).

**Methods:**

A retrospective cohort study of T2DM patients who were new users of sulfonylurea or metformin was conducted using the Yinzhou Regional Health Care Database. A marginal structural Cox model was used to estimate the hazard ratio (HR) of cancer associated with the use of sulfonylurea compared with metformin, with time-varying confounders controlled by inverse probability weighting. Secondary analyses using different glucose-lowering drugs (GLDs) as comparator and sensitivity analyses for potential bias due to latency period, model misspecification, missing data, analyses strategy (intention-to-treat and per-protocol), and diagnosis validation were performed to examine the robustness of the results.

**Results:**

After fully controlling for time-varying confounding, baseline confounding, and competing risk, the use of sulfonylurea was not associated with the risk of any cancer (HR 1.09; 95% CI, 0.93–1.27), compared with the use of metformin. In the secondary analyses, compared with α - glucosidase inhibitors, thiazolidinediones, glinides, other GLDs except sulfonylure and insulin, and T2DM patients not treated with sulfonylureas, the HRs of the association between sulfonylurea use and cancer risk were 0.92 (95% CI; 0.78–1.08), 0.89 (95% CI; 0.66–1.19), 0.85 (95% CI; 0.71–1.02), 1.04 (95% CI; 0.89–1.22), and 1.07 (95% CI; 0.99–1.16), respectively. The results of analyses for various subgroups, risk of site-specific cancers, cumulative duration, dose-response relationship, and sensitivity analyses of different latency periods and missing data were generally consistent with the findings of the primary analyses.

**Conclusion:**

No association between sulfonylurea use and cancer risk was found in this study after properly controlling biases due to time-varying confounders and other sources. Further studies on the association between sulfonylurea use and the risk of cancer by using data from a Chinese population with higher representativeness are needed.

## Introduction

Diabetes and cancer have been major health issues worldwide, with nearly half a billion people living with diabetes (1) and cancer being the first or second leading cause of death in over 100 countries (2). These diseases are of particular concern in China, where a quarter of world’s diabetic patients live (1). Further, 24% of newly diagnosed cancers and 30% of cancer-related deaths worldwide occur in China (3). Epidemiologic studies have suggested that diabetes can increase the risk of cancers and some observational studies have shown that glucose-lowering drugs (GLDs) can also affect this risk (4), raising concerns about the safety of GLDs.

Sulfonylurea is one kind of the most commonly used GLDs and has been used in clinical settings for more than 60 years. In China and some developed countries, the use of sulfonylureas is second only to or even exceeds the first-line oral GLD metformin (5, 6). Although a large number of observational studies about the association between GLDs and cancer incidence have been conducted, evidence of the association between sulfonylurea and cancer risk is still highly controversial (4, 7). Besides the diversity of study populations, the majority of these studies suffered from severe methodological limitations, such as immortal time bias and time-varying confounding, which have contributed to the current inconsistent findings (4, 7). Further, time-varying confounders affected by previous treatment is likely to be an important issue in this context because sulfonylurea use for type 2 diabetes mellitus (T2DM) is influenced by past disease severity, thus can change through time and in turn, sulfonylurea use can affect the status of glycemic control. For instance, glycated hemoglobin (HbA1c), a widely used measure of glycemic control, is an important predictor of drug initiation and modification for T2DM (8). The use of GLDs, including sulfonylurea, is likely to reduce future HbA1c. Also, evidence exists that HbA1c has effects on cancer risk (9). In this situation, HbA1c is a confounder between sulfonylurea use and cancer incidence but is also on the drug-cancer causal pathway. Conventional statistical models will fail to control this kind of time-varying confounder and result in biased estimation of the causal association (10). In addition, despite the increasing burden of diabetes and cancer, few studies focusing on the association between GLDs and cancer risk have been conducted in the mainland Chinese population.

Given the conflicting evidence of the sulfonylurea-cancer association and scarce evidence from the Chinese population, we carried out a population-based cohort study to assess the effects of sulfonylurea on the risk of cancer among T2DM patients. To reduce methodological flaws in previous studies, we evaluated the sulfonylurea-cancer association by applying the active-comparator new-user design (ACNU), in which patients initiating drug treatment of interest are compared with new users of another agent commonly used for the same indication, rather than with no treatment (nonuser group) (11). This principle helps to ensure similar treatment indications in treatment groups, mitigating both measured and unmeasured confounding by indication and immortal time bias (11, 12). Marginal structure models (MSMs) were used to control potential time-varying confounders, including HbA1c, body mass index (BMI), and other diabetes medications (13).

## Methods

### Data Source and Participants

We conducted a retrospective new-user active-comparator cohort study using the Yinzhou Regional Health Care Database (YRHCD). The YRHCD integrated longitudinal information of population census, electronic medical records, disease surveillance and management, health check, death registry, and other healthcare services in the Yinzhou District, Ningbo City of China (14, 15). Since 2009, the YRHCD has covered nearly all health-related activities of all residents in this region, from birth to death (14). In 2008, disease registry and management systems were established for patients of diabetes mellitus, cancer, cardiovascular disease, hypertension, and chronic obstructive pulmonary disease (14, 16). For diabetes patients, once registered, community physicians would follow up the patients at least four times a year, with common health measures, including blood pressure, fast blood glucose (FBG), HbA1c, smoking and drinking status being asked about or measured (16). The data used in this study and their relationship are presented in the (**Supplementary Figure S1**).

T2DM patients who were first diagnosed after January 1, 2009 and at least 18 years of age at diagnosis, were included in this study. Within this population, cohorts of new users of sulfonylurea or metformin were assembled. New users were identified by using a baseline washout period of 12 months before the first fill of sulfonylurea or metformin, during which the participants could not have prescription records of either drug. The date of the first fill was defined as the index date. Participants who initiated combination treatment of sulfonylurea and metformin at the index date and who had received a diagnosis of any cancer before the index date were excluded from the study. We further excluded patients having only one prescription of sulfonylurea or metformin to ensure that patients were actually started on these drugs.

The study was approved by the ethical review board of Peking University Health Science Center (approval number: IRB00001052-18013-Exempt).

### Exposure, Outcome, and Follow-Up

The exposure of sulfonylurea and the use of metformin were defined according to the outpatient and inpatient prescriptions and medication information in the disease registry and management system. Exposure was time-updated at the interval of six months and was defined in an as-treated manner based on actual drug use during follow-up. Exposure status was changed when participants switched between treatments. Discontinuation of drug use was defined as no further refill of sulfonylurea and metformin within six months of the previous prescription plus a 180-day grace period. Augmentation was defined as the addition of a comparator drug or combination of sulfonylurea and metformin.

The primary outcome was the incident diagnosis of any cancer, which was registered in the cancer registry and management system of the YRHCD. In addition, those who were not in the cancer registry system but had at least two inpatient or outpatient visits for the same cancer diagnosis with ICD-10 codes C00-C96 within one year were also considered cancer cases. The date of the first diagnosis was defined as the outcome date. Because cancer has a long preclinical phase, we assumed a six-month latency period for cancer pathogenesis in the primary analysis. All cancer cases diagnosed within six months after the index date were excluded.

We used six-month periods for assessing follow-up and time-updated exposure and covariates at the beginning of each new period. Participants were followed-up from the index date until the first occurrence of the following events: diagnosis of any cancer, death, drop-out (no any follow-up or medical records within six months from the last follow-up), six months of the carry-over period after discontinuation of sulfonylurea and metformin, augmentation or combination therapy, or the end of the study period (October 31, 2020).

### Covariates

Covariates included both time-invariant and time-varying factors that may influence the selection of GLDs and cancer risk. The time-invariant factors were measured in the baseline washout period and included age, gender, education level, smoking and drinking behavior, and duration of T2DM at the index date. All other factors were time-varying and were measured every six months, including other antidiabetic drugs except sulfonylurea and metformin (α-glucosidase inhibitors, thiazolidinediones [TZDs], Dipeptidyl peptidase 4 (DPP-4) inhibitors, glinides, and insulin), common medications for cardiovascular diseases (diuretics, beta-blocking agents, calcium channel blockers, angiotensin-converting enzyme inhibitors (ACEI), angiotensin receptor blockers (ARB), and aspirin), commonly used antibiotics (penicillins, cephalosporins, macrolides, quinolones, and other antibiotics), statins, and proton-pump inhibitors (PPI). Further, blood glucose level (fasting blood glucose (FBG) and HbA1c), blood lipid level and blood pressure, Charlson comorbidity index (CCI, calculated according to 14 kinds of potential comorbidities), BMI, healthcare utilization (hospitalizations and outpatient visits in prior six months) were included. Detailed definition of these covariates is presented in the (**Supplementary Table S1**).

### Statistical Analyses

Descriptive statistics summarized baseline covariates and standardized mean difference (SMD), which was not influenced by sample size and was used for comparisons between the two groups as suggested by Austin et al. (17). A SMD larger than 0.2 was used to show significant difference in the covariates (18). Marginal structural Cox models (MSCMs) with inverse probability weighting (IPW) were applied in the primary analyses for controlling potential time-varying confounders, which, such as HbA1c, could both predict subsequent exposure and be affected by past exposure history (10). It is an analytic challenge to address this kind of time-varying confounding, as conventional methods may block some of the treatment effects and induce collider-stratification bias at the same time (19). A pseudo-population in which time-varying covariates were independent of treatment assignment was created by implementing IPW, thus arriving at the causal association without bias (10). Pooled logistic regression models were fitted to estimate stabilized inverse probability of treatment weighting (IPTW) for each subject. The denominator of the IPTW was the cumulative probability of current exposure status condition on treatment history, time-varying and time-invariant confounders, and time since index date, modeled as a restricted cubic spline with knots at 0, 12, 24, 36, and 48 months. The numerator of the IPTW was estimated through a similar method but without time-varying confounders (20). To further control potential selection bias due to loss to follow-up, we also calculated the stabilized inverse probability of censoring weights (IPCWs) for different reasons of censoring: all-cause death, drop-out, treatment discontinuation, and treatment augmentation. IPCWs were the inverse of the probabilities of remaining uncensored at each follow-up, using a similar approach for the IPTW described above. The final stabilized IPW for each assessment was the product of the IPTW and IPCWs of different types of censoring and was truncated at the 99th percentile. Finally, the effect of sulfonylurea use on cancer risk was estimated using a weighted Cox model as a previous study (20). Another three standard Cox models with different confounding adjustment strategies were provided for comparison: unadjusted, baseline covariates adjusted, baseline and time-varying covariates adjusted. Multiple imputation was applied for imputing missing data using the full conditional specification method with five imputations according to the Quadratic Rule recommended by von Hippel (21).

We next examined the association of sulfonylureas and cancer risk within different subgroups for checking potential heterogeneity in subpopulations: age (<60 and ≥60 years), gender (female and male), CCI (0 and ≥1), smoking and drinking behavior, FBG (<7mmol/L and ≥7mmol/L), HbA1c (<7% and ≥7%) and duration of diabetes at the index date (<6 and ≥6 months). Cut-off points for FBG and HbA1c were defined according to the Chinese guidelines for the prevention and treatment of T2DM (22). Furthermore, the same MSCM model as the primary analyses was used for estimating the effects of sulfonylureas on the risk of site-specific cancer: gastric (ICD-10 code, C16), colorectal (C18-C21), liver (C22), pancreas (C25), lung (C34), breast (C50), prostate (C61), bladder (C67), thyroid (C73), lymphoma and leukemia (C81-C96), and all other cancers (ICD-10 code C00-C99 except the codes listed above). Association between cumulative years of sulfonylurea use and cancer risk was assessed by using MSCM and restricted cubic spline of the duration with five knots at the 0, 5%, 25%, 75%, and 95% percentile of the cumulative exposure years were applied for the potential non-linear dose-response relationship (23). The dose-response curve representing the association between the continuous exposure and cancer risk was presented using the method proposed by Desquilbeta (23).

### Secondary Analyses

We performed five prespecified secondary analyses using different comparators: α-glucosidase inhibitors, TZDs, glinides, all other glucose-lowering drugs except sulfonylureas and insulins (OGLD), and T2DM patients who were not treated with sulfonylurea. The comparator cohort was created following the same procedure as the primary analysis. DPP-4 inhibitors, glucagon-like peptide-1 (GLP-1) analogs, and sodium-glucose co-transporter 2 (SGLT2) inhibitors were not considered as a comparator because few T2DM patients were prescribed these drugs before 2018 in the study population (**Supplementary Figure S2**). Insulins were not used as a comparator because new users of insulin might have higher disease severity and induce indication confounding. MSCMs and standard Cox models similar to the primary analysis were applied in the secondary analyses.

Because multiple comparisons in the analyses of subgroups, site-specific cancers, and secondary analyses may increase the risk of type I error, findings of our secondary analyses should be interpreted as exploratory.

### Sensitivity Analyses

A series of sensitivity analyses were performed to examine the robustness of the results of the primary analysis. First, MSCM with no truncation on the weights was applied. Second, parametric g-formula, another kind of G-method, which can also properly deal with time-varying confounders affected by past exposure (19), was performed to repeat the primary analysis. Nonparametric bootstrap method was applied for the 95% confidence interval (CI). Third, the Fine-Gray subdistribution hazard model was used to check possible competing risk by death from any cause. Then, we repeated the primary analysis by varying the latency period to 0, 12, 18, and 24 months to evaluate the robustness of the assumptions of the latency time window. Another series of sensitivity analyses with follow-up starting at index date and different latency periods were conducted to further examine the results’ robustness. In addition, to evaluate the impact of missing data, we repeated the primary analysis after excluding subjects missing FBG or HbA1c at baseline. Furthermore, we performed our analyses in an intention-to-treat (ITT) and per-protocol (PP) fashion. In the ITT analysis, exposure status was determined according to the drug use at the index date and was not censored when treatment changed (switching to or augmentation with a comparator drug or discontinuation of the initiation drug). In the PP analysis, participants were further censored when switching between sulfonylurea and metformin. Furthermore, in order to enhance diagnostic accuracy of cancers, cancer case patients were required to be hospitalized for any anti-cancer therapy within one year of their first diagnosis and we conducted sensitivity analysis for this new definition of outcome. Finally, E-value, which was the minimum strength of association, that an unmeasured confounder would need to have with both the exposure and the outcome, to fully explain away an exposure outcome association (24) of positive results, was reported for assessing the robustness of our results against potential unmeasured confounding (24).

## Results

**Figure 1:**
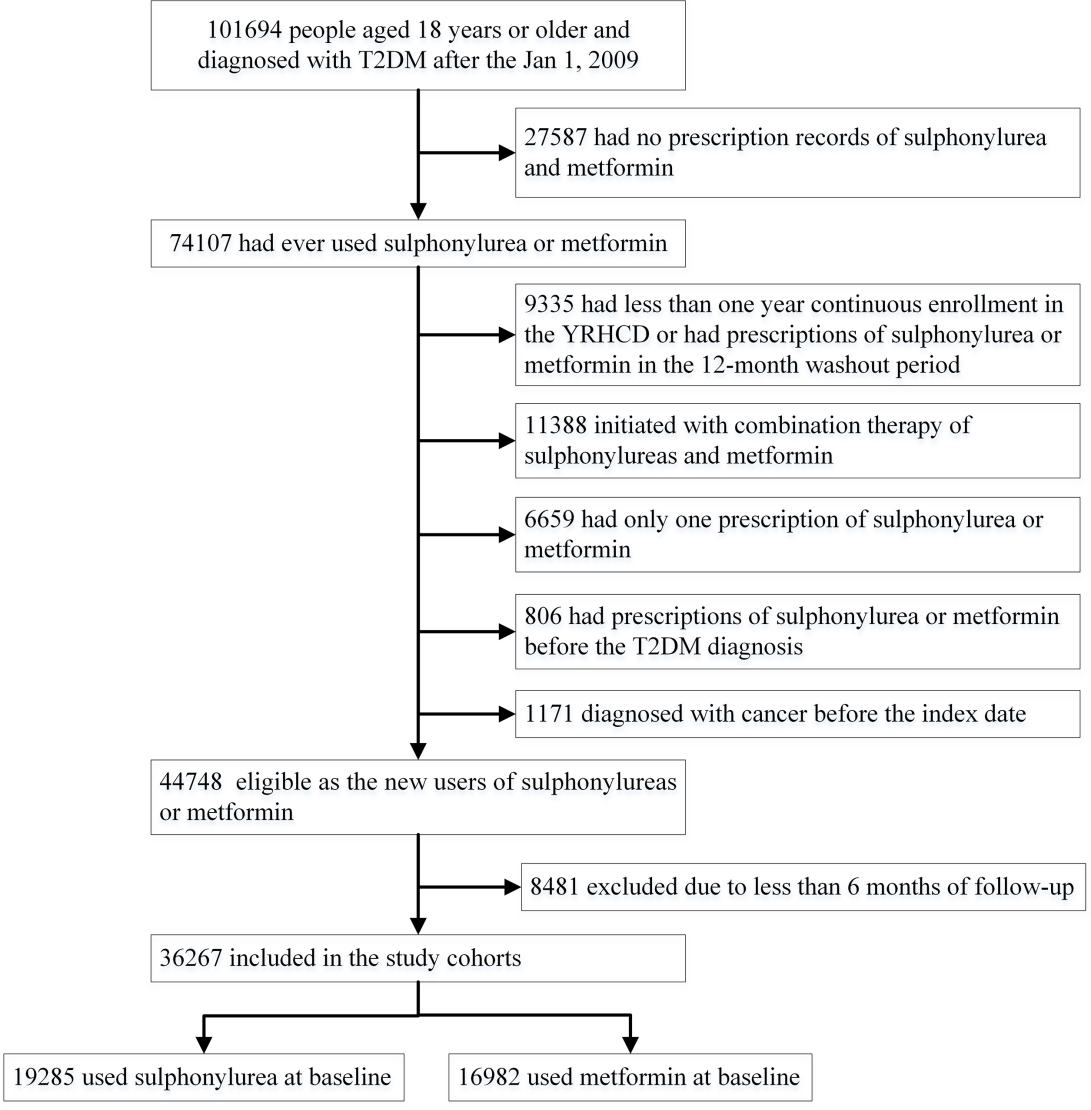
Flowchart of participants in the study cohorts. T2DM, type 2 diabetes mellitus.

In total, 101,694 T2DM patients aged 18 years or older who were first diagnosed after January 2009 were identified in the YRHCD (**Figure 1**). Sulfonylurea and metformin were the most commonly used GLDs in the study population, accounting for 35.2% and 28.5% of all GLD prescriptions (**Supplementary Figure S2**). The final analyses included 36,267 T2DM patients with a median of 1.9 years (interquartile range [IQR] 1.0–3.6 years, maximum follow-up 12 years). Of these participants, 19,285 initiated sulfonylurea at baseline and 16,982 were new metformin users (**Figure 1**). The median duration of follow-up for participants initiating sulfonylurea and metformin was 2.0 (IQR 1.0–3.9, maximum 12) and 1.8 (IQR 1.0–3.3, maximum 12) years, respectively. **Table 1** presents baseline characteristics for the entire cohort and stratified by sulfonylurea and metformin use at the index date. Overall, new users of sulfonylurea and metformin were similar for gender, smoking and drinking behavior, the prevalence of comorbidities, healthcare utilization, medication use for common chronic diseases, blood glucose and lipid levels, and blood pressure. However, compared with metformin new users, sulfonylurea users were older and less likely to have higher education. In addition, sulfonylurea users were less likely to be overweight or obese (BMI>24, 46.9% and 51.7%, respectively) and use insulin (3.0% and 5.4%, respectively) at baseline, but the differences were not significant. A total of 3,348 cancer cases occurred among all T2DM patients and the incidences of various types of cancers are given in the (**Supplementary Table S2**).

**Table 1:** Baseline characteristics of participants initiating sulfonylurea and metformin*.

	Overall	Sulfonylureas	Metformin	SMD
Age	59.6 (12.1)	61.0 (11.8)	58.1 (12.2)	0.239
Age≥60 years	18386 (50.7)	10471 (54.3)	7915 (46.6)	0.154
Male	18874 (52.0)	9838 (51.0)	9036 (53.2)	0.044
Education				
Senior high school or higher	5137 (14.2)	1998 (10.4)	3139 (18.5)	0.251
Junior high school	10746 (29.6)	5611 (29.1)	5135 (30.2)	
Primary school	13207 (36.4)	7612 (39.5)	5595 (32.9)	
Others	7177 (19.8)	4065 (21.1)	3113 (18.3)	
Smoking	11731 (32.3)	6234 (32.3)	5497 (32.4)	0.001
Drinking	12386 (34.2)	6590 (34.2)	5796 (34.1)	0.001
BMI(kg/m^2^)				
<18.5	679 (1.9)	406 (2.1)	273 (1.6)	0.112
[18.5,24)	17764 (49.0)	9839 (51.0)	7925 (46.7)	
[24,28)	14143 (39.0)	7300 (37.9)	6843 (40.3)	
≥28	3681 (10.1)	1740 (9.0)	1941 (11.4)	
Charlson comorbidity index				
0	28668 (79.0)	15259 (79.1)	13409 (79.0)	0.020
1	5196 (14.3)	2774 (14.4)	2422 (14.3)	
2	1683 (4.6)	866 (4.5)	817 (4.8)	
3	476 (1.3)	263 (1.4)	213 (1.3)	
≥4	244 (0.7)	123 (0.6)	121 (0.7)	
Inpatient admissions				
0	34190 (94.3)	18201 (94.4)	15989 (94.2)	0.031
1	1854 (5.1)	948 (4.9)	906 (5.3)	
≥2	223 (0.6)	136 (0.7)	87 (0.5)	
Outpatient visits				
0	8407 (23.2)	4423 (22.9)	3984 (23.5)	0.050
1~6	15066 (41.5)	7914 (41.0)	7152 (42.1)	
7~12	7436 (20.5)	3945 (20.5)	3491 (20.6)	
13~18	3044 (8.4)	1684 (8.7)	1360 (8.0)	
>18	2314 (6.4)	1319 (6.8)	995 (5.9)	
Duration of T2DM ≥ 6 months	18727(51.6)	10080 (52.3)	8647(50.9)	0.027
Medication use				
Insulin	1499 (4.1)	574 (3.0)	925 (5.4)	0.123
α-glucosidase inhibitors	3295 (9.1)	1808 (9.4)	1487 (8.8)	0.022
TZDs	885 (2.4)	521 (2.7)	364 (2.1)	0.036
DPP-4i	327 (0.9)	115 (0.6)	212 (1.2)	0.068
Glinides	1558 (4.3)	663 (3.4)	895 (5.3)	0.090
Diuretics	5427 (15.0)	2969 (15.4)	2458 (14.5)	0.026
Beta blocking agents	3429 (9.5)	1734 (9.0)	1695 (10.0)	0.034
Calcium channel blockers	10776 (29.7)	5667 (29.4)	5109 (30.1)	0.015
ACEI	2411 (6.6)	1379 (7.2)	1032 (6.1)	0.043
ARB	10547 (29.1)	5548 (28.8)	4999 (29.4)	0.015
Statins	5176 (14.3)	2627 (13.6)	2549 (15.0)	0.040
Aspirin	3181 (8.8)	1663 (8.6)	1518 (8.9)	0.011
PPI	5152 (14.2)	2903 (15.1)	2249 (13.2)	0.052
Penicillins	2497 (6.9)	1472 (7.6)	1025 (6.0)	0.063
Cephalosporins	7924 (21.8)	4336 (22.5)	3588 (21.1)	0.033
Macrolides	3375 (9.3)	1850 (9.6)	1525 (9.0)	0.021
Quinolones	3905 (10.8)	2116 (11.0)	1789 (10.5)	0.014
Other antibiotics	3383 (9.3)	1903 (9.9)	1480 (8.7)	0.040
FBG≥7mmol/L	20921 (57.7)	11114 (57.6)	9807 (57.7)	0.002
HbA1c≥7%	21770 (60.0)	11659 (60.5)	10112 (59.5)	0.019
FBG (mmol/L, log)	2.0 (0.2287)	2.0 (0.2278)	2.0 (0.2297)	0.011
HbA1c (%, log)	2.0 (0.2291)	2.0 (0.2291)	2.0 (0.2291)	0.014
HDLC (mmol/L, log)	0.2 (0.2498)	0.2 (0.2492)	0.2 (0.2503)	0.017
LDLC (mmol/L, log)	1.0 (0.3243)	1.0 (0.3255)	1.0 (0.3229)	0.016
TC (mmol/L, log)	1.6 (0.2249)	1.6 (0.2248)	1.6 (0.2250)	0.008
TG (mmol/L, log)	0.5 (0.5162)	0.4 (0.5142)	0.5 (0.5170)	0.109
SBP (mmHg, log)	4.8 (0.0616)	4.8 (0.0620)	4.8 (0.0611)	0.074
DBP (mmHg, log)	4.4 (0.0714)	4.4 (0.0718)	4.3 (0.0710)	0.041

*For continuous variables, the values are mean (standard deviation); for categorical variables the values are number (percentage). Definitions of all covariates are given in the **Supplementary Table S1**.

T2DM, type 2 diabetes mellitus; TZD, thiazolidinediones; DPP-4i, dipeptidyl peptidase-4 inhibitors; ACEI, angiotensin-converting enzyme inhibitors; ARB, angiotensin receptor blockers; PPI, proton-pump inhibitors; FBG, fast blood glucose; HbA1c, glycated haemoglobin; HDLC, high-density lipoprotein cholesterol; LDLC, low-density lipoprotein cholesterol; TC, total cholesterol; TG, triglyceride; SBP, systolic blood pressure; DBP, diastolic blood pressure.

**Table 2** shows the results of primary and secondary analyses. The cancer incidences among users of sulfonylurea and metformin were 847 and 726 per 100,000 person-years, respectively. Compared with metformin use, the crude analysis indicated that sulfonylurea use was associated with an increase risk of cancer (HR 1.23; 95%CI 1.06–1.44). However, after adjusting potential baseline and time-varying confounding, the result of MSCM presented that use of sulfonylurea was not associated with cancer incidence (HR 1.09; 95%CI, 0.93–1.27). The mean weight of MSCM was 1.006, and the median was 0.962 (IQR, 0.856–1.095). A IPW weighted Kaplan-Meier survival curve is given in the (**(Supplementary Figure S3**). Results of the standard Cox models were consistent with the MSCM. Furthermore, all results of the secondary analyses were consistent and showed that there was no significant association between sulfonylurea use and cancer risk. The HRs of the associations between use of sulfonylurea and risk of cancer were 0.92 (95%CI, 0.78–1.08), 0.89 (95% CI, 0.66–1.19), 0.85 (95% CI, 0.71–1.02), 1.04 (95% CI, 0.89–1.22), and 1.07 (95% CI, 0.99–1.16), respectively, when compared with α-glucosidase inhibitors, TZDs, Glinides, OGLD, and T2DM patients who were not treated with sulfonylurea. Weight distributions of MSCMs for secondary analyses are given in the (**(Supplementary Table S3**).

**Table 2:** Association between the sulfonylurea use and risk of all cancers.

Comparators	Cancer cases/Follow-up years	Incidence (/100,000 person years)	Crude HR (95% CI)	Adjusted HR (95% CI) for baseline covariates	Adjusted HR (95% CI) for baseline and time-varying covariates	Adjusted HR (95% CI) from marginal structural model
**Metformin**						
Comparator	291/40082	726	Reference	Reference	Reference	Reference
Sulfonylureas	462/54514	847	1.23 (1.06–1.44)	1.10 (0.94–1.29)	1.09 (0.93–1.28)	1.09 (0.93–1.27)
**α-glucosidase inhibitors**						
Comparator	228/26926	847	Reference	Reference	Reference	Reference
Sulfonylureas	670/89565	748	0.93 (0.79–1.09)	0.97 (0.82–1.14)	0.97 (0.82–1.15)	0.92 (0.78–1.08)
**TZDs**						
Comparator	68/7282	934	Reference	Reference	Reference	Reference
Sulfonylureas	805/110909	726	0.99 (0.75–1.32)	0.95 (0.71–1.26)	0.96 (0.72–1.28)	0.89 (0.66–1.19)
**Glinides**						
Comparator	165/17065	967	Reference	Reference	Reference	Reference
Sulfonylureas	902/122753	735	0.88 (0.73–1.05)	0.86 (0.72–1.04)	0.87 (0.73–1.05)	0.85 (0.71–1.02)
**All other glucose-lowering drugs except sulfonylurea and insulins**						
Comparator	485/63526	763	Reference	Reference	Reference	Reference
Sulfonylureas	285/32287	883	1.11 (0.95–1.29)	1.03 (0.89–1.21)	1.04 (0.89–1.21)	1.04 (0.89–1.22)
**T2DM patients that were not treated with sulfonylurea**						
Comparator	2427/251818	964	Reference	Reference	Reference	Reference
Sulfonylureas	1563/143280	1091	1.13 (1.04–1.22)	1.08 (1.00–1.17)	1.07 (0.99–1.16)	1.07 (0.99–1.16)

**Figure 2:**
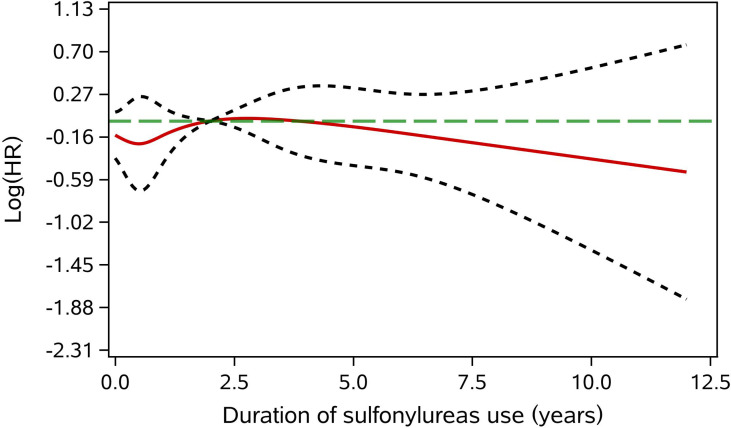
Adjusted dose-response association between cumulative duration of sulfonylurea use and cancer risk. The green long dotted line is the null value (HR = 1). The red solid line represents the point estimate of the log(HR). The two gray short dash line are the limits of 95%CI. The reference value was the median of cumulative duration of sulfonylurea use.

Subgroup analyses found no association between the use of sulfonylurea and cancer risk in different subpopulations of T2DM patients defined by baseline characteristics (**Table 3**). Within different subgroups, the adjusted HRs of MSCMs ranged between 0.99 and 1.34, with all confidence intervals contained the null value (HR=1). In addition, further analyses suggested that there were no associations between use of sulfonylurea and risk of site-specific cancers except lymphoma and leukemia (**Table 4**), of which the results showed that sulfonylurea use might be associated with an increased risk (HR 2.23; 95% CI, 1.04–4.76). However, the E-value for the lower limit of the CI was just 1.2. The analysis of cumulative duration of sulfonylurea use suggested that there was no dose-response relationship in the association between sulfonylurea exposure and cancer risk (**Figure 2**).

**Table 3:** Association between the use of sulfonylurea and the risk of any cancer in various subgroups of participants.

	Sulfonylureas		Metformin		HR (95% CI)
	Cases/Person-years	Incidence (/100,000 person years)	Cases/Person-years	Incidence (/100,000 person years)	
**Age group**					
<60 years	138/24417	565	99/21088	469	1.14 (0.86–1.50)
≥60 years	324/30096	1077	192/18994	1011	1.08 (0.90–1.30)
**Gender**					
Female	206/27397	752	122/19078	639	1.21 (0.95–1.54)
Male	256/27117	944	169/21004	805	1.03 (0.84–1.26)
**Drinking**					
No	301/35430	850	187/25764	726	1.08 (0.89–1.31)
Yes	161/19084	844	104/14318	726	1.09 (0.84–1.42)
**Smoking**					
No	270/37113	728	178/26828	663	1.01 (0.83–1.24)
Yes	192/17401	1103	113/13254	853	1.23 (0.96–1.58)
**FBG**					
<7mmol/L	221/23269	950	140/17380	803	1.14 (0.90–1.45)
≥7mmol/L	241/31244	771	151/22702	667	1.05 (0.83–1.33)
**HBA1c**					
<7%	197/21862	900	125/16356	767	1.10 (0.74–1.63)
≥7%	265/32651	813	166/23727	698	1.08 (0.80–1.46)
**Duration of T2DM**					
<0.5 years	210/25683	818	142/18547	766	0.99 (0.79–1.24)
≥0.5 years	252/28831	874	149/21535	692	1.17 (0.95–1.45)
**CCI**					
0	346/44269	782	230/32842	700	1.03 (0.87–1.23)
≥1	116/10244	1132	61/7240	843	1.34 (0.96–1.86)

**Table 4:** Association between the use of sulfonylurea and the risk of site-specific cancer.

	Sulfonylureas (54513.5 person-years)		Metformin (40082.1 person-years)		HR (95% CI)
	Cases	Incidence (/100,000 person years)	Cases	Incidence /100,000 person years)	
Gastric cancer	72	132	45	112	0.98 (0.67–1.43)
Colorectal cancer	45	83	26	65	1.09 (0.67–1.77)
Liver cancer	26	48	24	60	0.78 (0.43–1.41)
Pancreas cancer	18	33	16	40	0.94 (0.46–1.96)
Lung cancer	94	172	50	125	1.19 (0.82–1.71)
Breast cancer	27	50	19	47	0.89 (0.46–1.73)
Prostate cancer	26	48	16	40	0.87 (0.46–1.68)
Bladder cancer	15	28	9	22	1.68 (0.64–4.40)
Thyroid cancer	25	46	22	55	0.82 (0.45-1.50)
Lymphoma & leukemia	29	53	11	27	2.23 (1.04–4.76)
All other cancers	85	156	53	132	1.26 (0.86–1.82)

Series of sensitivity analyses yielded consistent results with that of the primary analysis (**Table 5**). In the ITT analyses, participants were followed for a median of 4.8 years (IQR, 2.8–7.2) and 3.8 years (IQR, 2.0–6.5) in T2DM patients initiating sulfonylurea and metformin, respectively. However, no association between the use of sulfonylurea and cancer risk was observed in the analyses of various models and different latency period settings, excluding participants missing FBG and HbA1c and alternative analysis strategies of ITT and PP. In addition, sulfonylurea use was not significantly associated with increased cancer risk (HR 1.15; 95% CI, 0.97–1.36) when the outcome was defined as cancer patients who were hospitalized for any anti-cancer therapy within one year of the first cancer diagnosis.

**Table 5:** Results of sensitivity analyses.

	Sulfonylureas		Metformin		HR (95% CI)
	Cases/Person-years	Incidence (/100,000 person years)	Cases/Person-years	Incidence (/100,000 person years)	
**Alternative models**					
Subdistribution hazard function	462/54514	847	291/40082	726	1.10 (0.94–1.28)
MSM with raw weights	462/54514	847	291/40082	726	1.09 (0.93–1.27)
Parametric g-formula	462/54514	847	291/40082	726	1.06 (0.89–1.27)
**Latency periods (months), follow-up from the index date**					
0 months	570/55437	1028	393/41064	957	1.04 (0.90–1.19)
12 months	383/51312	746	233/36962	630	1.11 (0.94–1.33)
18 months	311/47912	649	190/33535	567	1.08 (0.89–1.31)
24 months	262/44242	592	160/30055	532	1.07 (0.86–1.32)
**Latency periods (months), follow-up after the latency periods**					
6 months	455/45063	1010	278/31759	875	1.10 (0.94–1.28)
12 months	319/31780	1004	197/21234	928	1.11 (0.94–1.32)
18 months	303/29888	1014	172/19388	887	1.06 (0.87–1.28)
24 months	255/24342	1048	143/15257	937	1.05 (0.85–1.29)
**Excluding individuals missing FBG or HbA1c at baseline**	392/46678	840	265/33946	781	1.03 (0.87–1.21)
**Alternative analysis strategies**					
Intention-to-treat	908/115086	789	573/86985	659	1.07 (0.97–1.19)
Per-protocol	404/50206	805	233/37181	627	1.08 (0.91–1.27)
**Hospitalization within one year of cancer diagnosis**	410/54514	752	240/40082	599	1.15 (0.97–1.36)

## Discussion

In this population-based cohort study, sulfonylurea and metformin were the most frequently used diabetes medications, accounting for 64% of all GLDs in the study population. We found that sulfonylurea use was not associated with cancer risk compared with other GLDs. Our results were consistent across all secondary analyses using different GLDs as a comparator. Further, subgroup analyses, cumulative exposure duration, dose-response analysis, and sensitivity analyses were all in line with the main finding. In addition, despite the result of potentially increased risk of lymphoma and leukemia, the use of sulfonylurea was not associated with the risk of other site-specific cancers.

Our estimate of cancer incidence among T2DM patients was little higher than a previous study conducted by Pan et al, who estimated that the incidence of cancer in T2DM patients was 576.3/100000 person-years in China using data from the China Kadoorie Biobank, a large population-based prospective cohort covering 10 diverse regions of China (25), reflecting regional differences in cancer incidence. In this study, sulfonylurea was widely used as the first-line initial drug in the Yinzhou population, which was consistent with previous studies that found 30%–40% of newly diagnosed type 2 diabetes patients received sulfonylurea as initial treatment (5, 26).

Previous studies investigating the association between the use of sulfonylurea and cancer risk have provided conflicting results. Meta-analyses of RCTs showed no difference in cancer risk between the use of sulfonylurea and other GLDs (27, 28). Series of cohort studies suggested that the use of sulfonylurea may increase, decrease, or have no effect on the risk of cancer when compared with other GLDs or non-user of sulfonylurea (28–30). However, most of these observational studies may have flaws in study design and data analysis methods (7, 13). According to the systematic review conducted by Farmer (13), observational studies on comparing cancer risk between users of metformin and sulfonylurea varied in design, and the majority had risks of several kinds of bias due to time-varying confounding and other sources. In contrast, our results were consistent with the findings of studies that have low risk of bias due to time-varying confounding (13, 31, 32). In the cohort analysis using the U.K. Clinical Practice Research Datalink (CPRD), Tsilidis et al. found that sulfonylurea and metformin had a similar effect on the incidence of total cancer (31). In another study based on CPRD, van Staa et al. found that use of sulfonylurea was associated with an increased risk of cancer within 6 months of drug initiation compared with metformin (32). However, this association was not likely to be a causal effect but due to protopathic bias (32). Furthermore, two database cohort studies among the German and British populations found that sulfonylurea did not increase the risk of cancer (33, 34). In terms of the associations between use of sulfonylurea and risk of site-specific cancers, our results were consistent with most of the current studies except that we observed an increased risk of lymphoma and leukemia among users of sulfonylurea. For example, a series of recent studies found no association between sulfonylurea use and risk of breast cancer (31, 32, 35, 36) no matter whether these studies had a high risk of time-varying confounding and other sources of bias (13). However, current evidence of the association between sulfonylurea and the incidence of lymphoma and leukemia is very scarce. One study by Tsilidis et al. using the CPRD data found that sulfonylurea users had a non-significant higher risk of leukemia and non-Hodgkin lymphoma (31). Although our results suggest that sulfonylurea was potentially associated with the increased risk of lymphoma and leukemia, the E-value was just 1.2, meaning that the sulfonylurea-cancer association could be insignificant if there existed some unmeasured confounders (24). Thus, more studies are needed to further investigate the potential effect of sulfonylurea use on the risk of lymphoma and leukemia.

In this study, time-varying confounders that were also affected by past treatment did not seem to be a critical issue because the results of the conventional models and MSCMs were highly consistent after adjusting time-invariant and time-varying confounders. However, this similarity needs to be interpreted with caution. Because using conventional methods to control time-varying confounders affected by past treatment, such as HbA1c, will block some of the treatment effects (by conditioning on the time-varying confounders lying on the causal pathway between past treatment and outcome) and induce collider-stratification bias when such time-varying confounders are affected by unmeasured factors that predict the outcome (e.g., dietary habit was not included in this study) (19, 37). These two kinds of bias might cancel out because of the same magnitude but opposite direction, leading to an unbiased estimate and similar results to those of MSCMs (37). On the contrary, when these biases have different magnitude and/or the same direction, substantially biased estimates may occur in conventional methods. Two systematic reviews suggested that in about 40% of studies comparing methods properly dealing with time-varying confounding and conventional models, the results differed by at least 20%; and in 11% of these studies, the two methods resulted in estimates of opposite directions (38, 39). Therefore, MSMs or other methods that can properly control time-varying confounders, such as g-formula and structure nested model (19), should be used whenever this kind of confounding is likely to occur (38) as primary analysis or at least sensitivity analysis.

Our study had several strengths. First, we applied the ACNU design, avoiding immortal-time bias and minimizing confounding by indications through adjusting various potential confounders (11, 12). Second, we evaluated the association between the use of sulfonylurea and cancer risk compared with different kinds of GLDs in a series of secondary analyses, of which the results were consistent with the primary analysis, thus enhancing the reliability of our results. Third, we conducted a series of sensitivity analyses for the latency period, model specification, missing data, analysis strategy, and diagnosis validation. The findings were consistent across all sensitivity analyses. Fourth, results of various analyses of different subgroups, effects of sulfonylurea use on the risk of site-specific cancers, and cumulative exposure duration and potential dose-response relationship, were generally in concordance with the main finding. Finally, we applied MSCMs and g-formula to deal with potential time-varying confounders affected by past treatment, which was rarely considered in previous studies of the glucose-lowering drug-cancer association (13). Although no substantial differences between results of MSCMs and conventional Cox models were observed in this study, bias induced by time-varying confounding should be considered whenever possible because it can have a crucial impact on the association estimate in specific circumstances (38).

This study had some limitations. First, the study population was from a single municipal district in China, where the incidence of cancers may vary greatly in different regions. A limited number of cancer events were observed in the study population, especially for some rare cancers, such as pancreas cancer and adult lymphoma and leukemia, thus, extrapolating our findings to other populations should be made with caution. Further studies based on a population with higher representativeness are needed. Second, although quite a number of time-invariant and time-varying covariates were included in the analyses and ACUN design might have further decreased the bias caused by unmeasured confounding (12), some potential confounders, such as dietary patterns, were not adjusted due to lack of relevant information in the database. This might have some effect on the positive finding of this study because of its low E-value (24). Third, compared with sulfonylurea and metformin, much fewer T2DM patients used other GLDs, leading to a limited sample size of the control group and lower power in the secondary analyses using specific kind of glucose-lowering agents as the comparator (e.g., TZDs). Thus, more studies on the association between use of sulfonylurea and cancer risk compared with non-metformin glucose-lowering drugs are need in the future. Fourth, drug dosage information is not well documented in the database, making it impossible to calculate time-specific standardized doses for different drugs, which was why we used treatment duration as a measure of cumulative exposure in the dose-response analysis. Finally, the median follow-up time was only two years because of the high prevalence of censoring caused by the combination therapy of sulfonylurea and metformin. However, in the ITT analysis ignoring the augmentation of these two kinds of drugs, the median duration of follow-up was 4.5 years and the result was consistent with the primary analysis. More studies with longer follow-up times are needed.

## Conclusions

This population-based cohort study did not find any cancer risk except for leukaemia and lymphoma in people with T2DM receiving sulfonylurea treatment, after properly controlling biases of time-varying confounders and other sources. Given the limited number of cancer events, the findings of site-specific cancer warrant external validation in a larger population. Our findings were in line with previous studies that have low risk of bias and results from relevant meta-analyses of RCTs. Further studies about the association between the use of sulfonylurea and risk of cancer compared with other glucose-lowering agents, especially non-metformin drugs, based on a population with higher representativeness and longer follow-up time, are needed in China.

## Data Availability Statement

Data used in this study are available to the scientific community and the requests should be sent to the corresponding author.

## Ethics Statement

The studies involving human participants were reviewed and approved by Ethical Review Board of Peking University Health Science Center (approval number: IRB00001052-18013-Exempt). Written informed consent for participation was not required for this study in accordance with the national legislation and the institutional requirements.

## Author Contributions

Contributors: HZ and SZ conceived of and designed the work. HL and PS acquired the data. HZ analyzed the data. HZ drafted the manuscript. ZL and LZ critically revised the manuscript for important intellectual content. SZ, HL, and PS supervised the study. SZ obtained the funding. All authors were responsible for the interpretation of the data, and revised, and gave final approval of the manuscript.

## Funding

This work was supported by the National Natural Science Foundation of China (Grant No. 81973146). The funders had no role in the design and conduct of the study; the collection, management, analysis, and interpretation of the data; preparation, review, or approval of the manuscript; and decision to submit the manuscript for publication.

## Conflict of Interest

The authors declare that the research was conducted in the absence of any commercial or financial relationships that could be construed as a potential conflict of interest.

## Publisher’s Note

All claims expressed in this article are solely those of the authors and do not necessarily represent those of their affiliated organizations, or those of the publisher, the editors and the reviewers. Any product that may be evaluated in this article, or claim that may be made by its manufacturer, is not guaranteed or endorsed by the publisher.
