# Supplementary material for: Sulfonylurea and Cancer Risk Among Patients With Type 2 Diabetes: A Population-Based Cohort Study
Source: Front Endocrinol (Lausanne). 2022 Jun 30;13:874344. doi: 10.3389/fendo.2022.874344 (PMC9279619; doi:10.3389/fendo.2022.874344)
Supplement: Supplementary file 1 [file DataSheet_1.pdf]

Appendix Table of Contents

Figure S1. Data structure of the YRHCD.
Table S1. Details of potential confounders included in this study.
Figure S2. Trends of the proportions of different kinds of antidiabetic agents.
Table S2. Incidences of different types of cancers among T2DM patients in Yinzhou region.
Figure S3. Inverse probability of treatment and censoring weighted Kaplan-Meier curves of cancer risk for T2DM patients treated with sulfonylurea and metformin.
Table S3. Weight distribution of MSCMs in various analyses.

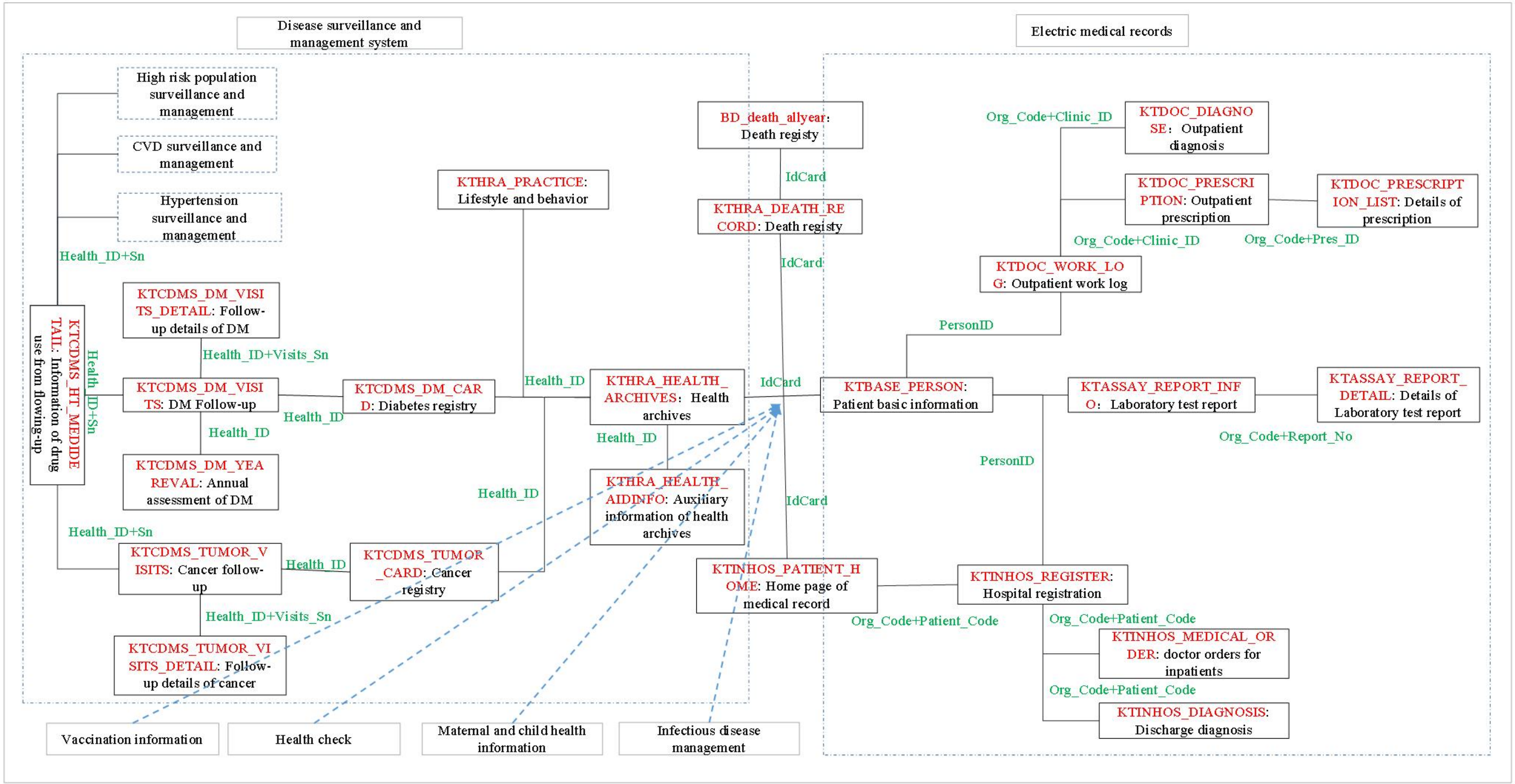

Figure S1. Data structure of the YRHCD.

Relationship of different tables is presented in the figure. Data from the disease surveillance and management and electric medical records were used in this study. Detailed information contained in each table in the figure and more details of other data can be found elsewhere.[1]

Red words are table names. Green words are keys for data linking.

We gave a more detail description of the data used in this study in the Table S2.

Table S1. Details of potential confounders included in this study

Covariates	Covariate types	Variable definition/transformation	Adjustment in the MSCMs[a]
**Demographic characteristics**			
Age	Continuous	≥18 years	spl / bv
Gender	Binary	Male and female	bin / bv
Education level	Categorical	Senior high school or higher, junior high school, primary school, and all others	cat / bv
**Concomitant medication**[b]		Defined according to ATC code.	
Otehr antidiabetic agents except sulfonylurea and comparator drug[b]	Binary	A group of covariates that had different definition. For instance, these covariates included α-glucosidase inhibitor, TZDs, DPP-4 inhibitor, glinides, and insulin.	bin / tv /lag-1
Diuretics[b]	Binary	C03	bin / tv /lag-1
Beta blocking agents[b]	Binary	C07	bin / tv /lag-1
Calcium channel blockers[b]	Binary	C08	bin / tv /lag-1
ACEI[b]	Binary	C09A	bin / tv /lag-1
ARB[b]	Binary	C09C	bin / tv /lag-1
Statins[b]	Binary	C10	bin / tv /lag-1
Penicillins[b]	Binary	J01C	bin / tv /lag-1
Cephalosporins[b]	Binary	J01D	bin / tv /lag-1
Macrolides[b]	Binary	J01F	bin / tv /lag-1
Quinolones[b]	Binary	J01M	bin / tv /lag-1
Other antibiotics[b]	Binary	Other drugs classified as J01 and not listed above	bin / tv /lag-1
Aspirin[b]	Binary	N02BA01、A10AD05、B10AC06	bin / tv /lag-1
PPI[b]	Binary	A02BC	bin / tv /lag-1
**Comorbidities** [c]		Defined according to ICD-10 code.	
CCI[c]	Categorical	0, 1, 2, 3, 4, and >4	cat / tv /lag-1
Myocardial infarction[d]	Binary*1	I21.x、I22.x、I25.2、I25.5	For calculating CCI, not included in the final analysis models
Congestive heart failure[d]	Binary*1	I11.0、I13.0、I13.2、I50	Ditto
Peripheral vascular disease[d]	Binary*1	I70、I71、I73.1、I73.8、I73.9、I77.1、I79.0、I792、K55.1、K55.8、K55.9、Z95.8、Z95.9	Ditto
Cerebrovascular disease[d]	Binary*1	G45、G46、H34.0、I60-I69	Ditto
Dementia[d]	Binary*1	F00-F03、F051、G30、G311	Ditto
Chronic pulmonary disease[d]	Binary*1	I278、I279、J40-J47、J60-J67、J684、J701、J703	Ditto
Connective tissue disease[d]	Binary*1	M05、M06、M31.5、M32、M33、M34、M35.1、M353、M36.0	Ditto
Ulcer disease[d]	Binary*1	K25-K28	Ditto

Mild liver disease[d]	Binary*1	B18、K70.0-K70.3、K709、K71.3-K71.5、K71.7、K73、K74、K76.0、 K76.2-K76.4、K76.8、K76.9、Z94.4	Ditto
Hemiplegia[d]	Binary*2	G041、G114、G801、G802、G81、G82、 G830、G831-G834、G839	Ditto
Diabetes with chronic complication[d]	Binary*2	E102-E105、E107、E112-E115、E117、E122-E125、E127、E132-E135、E137、E142-E145、E147	Ditto
Moderate or severe renal disease[d]	Binary*2	I120、I131、N032-N037、N052-N057、N18、N19、N250、Z49.0-Z49.2、Z940、Z992	Ditto
Moderate or severe liver disease[d]	Binary*3	I850、I859、I864、I982、K704、K711、 K721、K729、 K765-K767	Ditto
HIV/AIDS[d]	Binary*6	B20-B22、B24、Z21	Ditto
**Laboratory tests**			
FBG (mmol/L)	Continuous	Log transformation	spl / tv /lag-1
HbA1c (%)	Continuous	Log transformation	spl / tv /lag-1
HDLC (mmol/L)	Continuous	Log transformation	lin / tv /lag-1
LDLC (mmol/L)	Continuous	Log transformation	lin / tv /lag-1
TC (mmol/L)	Continuous	Log transformation	lin / tv /lag-1
TG (mmol/L)	Continuous	Log transformation	lin / tv /lag-1
**Physical examination**			
BMI (kg/m^2)	Categorical	<18.5, [18.5,24.0), [24.0,28.0) , and ≥28.0	cat / tv /lag-1
SBP (mmHg)	Continuous	Log transformation	lin / tv /lag-1
DBP (mmHg)	Continuous	Log transformation	lin / tv /lag-1
**Lifestyle**			
Smoking	Binary	Smoker, non-smoker	bin / bv
Drinking	Binary	Drinker, non-drinker	bin / bv
**Others**			
Duration of T2DM[e]	Binary	<0.5 years, ≥0.5years	bin / bv
Follow-up time (6-month interval)	Continuous	Months from the index date	spl / tv

[a] spl: Restricted cubic spline functions; cat: Categorical; bin: Binary; lin: linear; bv: baseline value or time-invariant covariates; tv: time-varying; lag-1: one-interval (six-month) lagged value of the variable was included in the models.

[b] Defined according to the Anatomical Therapeutic Chemical (ATC) Classification system. ACEI: Angiotensin converting enzyme inhibitors; ARB: Angiotensin II receptor blockers ; PPI: Proton-pump inhibitors.

[c] CCI: Charlson Comorbidity Index, defined according to 14 kinds of comorbidities.

[d] Defined according to the 10th version of International Classification of Disease (ICD-10). *w was the weight of the comorbidity when calculating the CCI. The third column contains the ICD-10 codes of the corresponding comorbidity.

[e] In the secondary analyses in which T2DM patients not treated with sulfonylureas were the control, duration of T2DM for all patients in the cohort was 0 years. Thus, this covariate was not included in the MSCM.

Abbreviations: T2DM: type 2 diabetes mellitus; TZD: thiazolidinediones; DPP-4i: dipeptidyl peptidase-4 inhibitors; ACEI: angiotensin-converting enzyme inhibitors; ARB: angiotensin receptor blockers; PPI: proton-pump inhibitors; FBG: fast blood glucose; HbA1c: glycated haemoglobin; HDLC: high-density lipoprotein cholesterol; LDLC: low-density lipoprotein cholesterol; TC: total cholesterol; TG: triglyceride; SBP: systolic blood pressure; DBP: diastolic blood pressure.

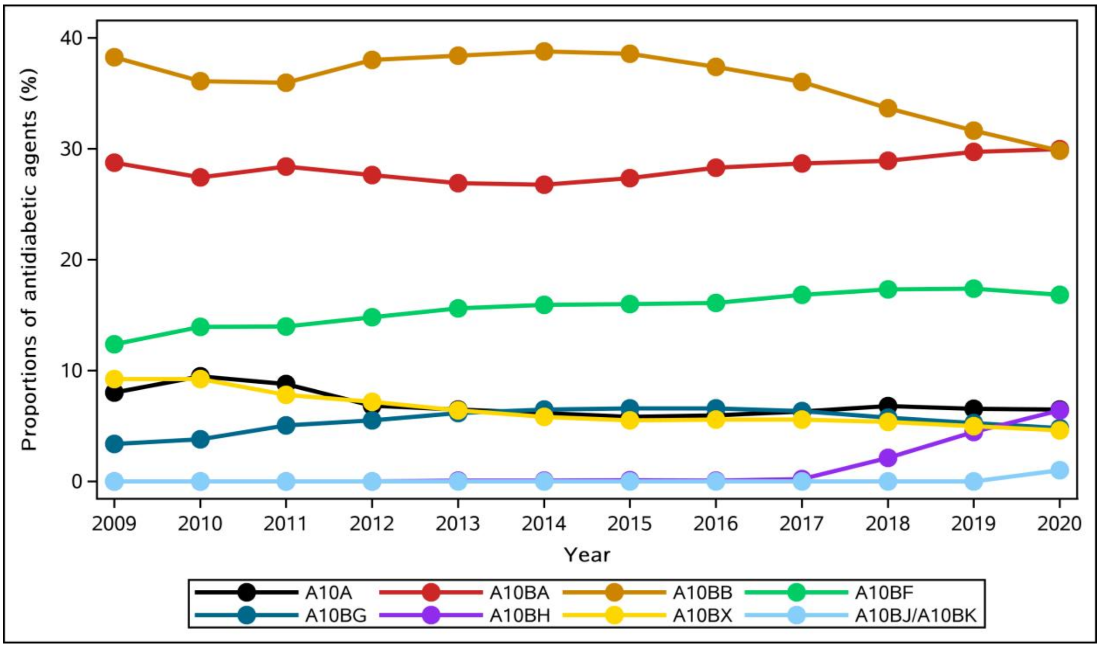

Figure S2. Trends of the proportions of different kinds of antidiabetic agents.

A10A: Insulins; A10BA: Metformin; A10BB: Sulfonylureas; A10BF: α-glucosidase inhibitors; A10BG: Thiazolidinediones; A10BH: Dipeptidyl peptidase-4 inhibitors; A10BJ: Glucagon-like peptide-1 analogues; A10BK: Sodium-glucose co-transporter 2 inhibitors.

Table S2 Incidences of different types of cancers among T2DM patients in Yinzhou region

	Male (245980.1 person-years)		Female （241759.6person-years）		Over all （487739.7person-years）	
	Cases	Incidence (/100000 PY)	Cases	Incidence (/100000 PY)	Cases	Incidence (/100000 PY)
All caners	1899	772 (737-807)	1449	599 (568-630)	3348	686 (663-710)
Gastric cancer	357	145 (130-160)	140	58 (48-68)	497	102 (93-111)
Colorectal cancer	224	91 (79-103)	154	64 (54-74)	378	78 (70-85)
Liver cancer	185	75 (64-86)	44	18 (13-24)	229	47 (41-53)
Pancreas cancer	73	30 (23-36)	51	21 (15-27)	124	25 (21-30)
Lung cancer	372	151 (136-167)	244	101 (88-114)	616	126 (116-136)
Breast cancer	5	2 (0-4)	217	90 (78-102)	222	46 (40-52)
Prostate cancer	159	65 (55-75)	—	—	159	33 (28-38)
Bladder cancer	74	30 (23-37)	24	10 (6-14)	98	20 (16-24)
Thyroid cancer	65	26 (20-33)	191	79 (68-90)	256	52 (46-59)
Lymphoma & leukemia	75	31 (24-37)	63	26 (20-32)	138	28 (24-33)
All other cancers	310	126 (112-140)	321	133 (118-147)	631	129 (119-139)

PY: person-yeras.

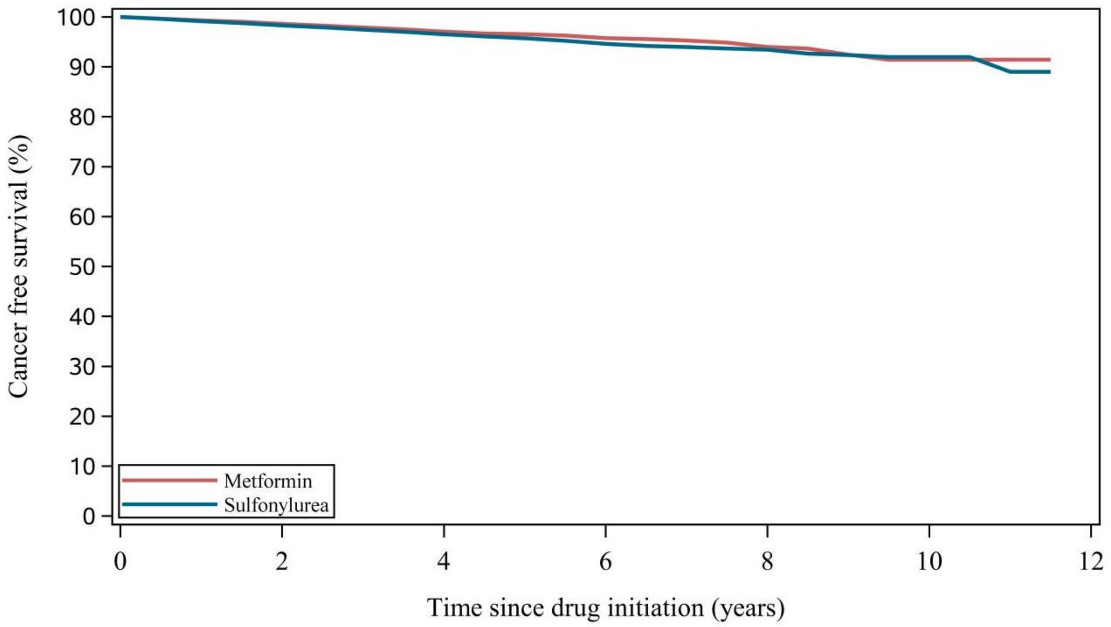

	Subjects	Cancer cases	Death	Dropout
Metformin	16982	291	136	4345
Sulfonylurea	19285	462	299	5446

Figure S3 Inverse probability of treatment and censoring weighted Kaplan-Meier curves of cancer risk for T2DM patients treated with sulfonylurea and metformin.

Table S3. Weight distribution of MSCMs in various analyses

Analyses	Mean (SD)	Median (IQR)	Range	1th and 99th percentiles
**Primary analysis**				
Metformin	1.006(0.285)	0.962(0.856-1.095)	0.112-22.008	0.558-1.975
**Secondary analyses**				
α-glucosidase inhibitors	1.004(0.386)	0.959(0.875-1.066)	0.055-67.084	0.435-2.086
TZDs	1.005(0.289)	0.976(0.922-1.034)	0.028-22.983	0.470-1.928
Glinides	1.004(0.275)	0.978(0.913-1.054)	0.082-19.997	0.492-1.873
OGLD	1.002(0.232)	0.977(0.884-1.081)	0.182-33.969	0.610-1.742
T2DM patients that were not treated with sulfonylureas	1.007(0.443)	0.967(0.839-1.087)	0.001-29.993	0.283-2.558
**Subgroup analyses**				
Age group (years)				
<60	1.009(0.288)	0.965(0.852-1.105)	0.122-9.411	0.544-2.010
≥60	1.006(0.297)	0.960(0.856-1.094)	0.144-16.858	0.543-1.992
Gender				
Female	1.009(0.289)	0.963(0.856-1.098)	0.111-9.694	0.543-2.047
Male	1.006(0.290)	0.961(0.851-1.102)	0.127-14.577	0.547-1.969
Drinking status				
Non dinker	1.006(0.277)	0.963(0.858-1.094)	0.118-19.164	0.558-1.974
Drinker	1.011(0.321)	0.962(0.848-1.106)	0.107-17.933	0.526-2.071
Smoking status				
Non smoker	1.007(0.272)	0.965(0.861-1.095)	0.108-9.867	0.561-1.968
Smoker	1.008(0.312)	0.958(0.843-1.106)	0.096-15.729	0.525-2.062
FBG at baseline (mmol/L)				
<7	1.008(0.276)	0.963(0.855-1.100)	0.097-11.762	0.571-1.950
≥7	1.008(0.320)	0.960(0.850-1.100)	0.105-12.273	0.517-2.085
HbA1c at baseline (%)				
<7	1.009(0.286)	0.966(0.858-1.100)	0.121-33.902	0.567-1.965
≥7	1.007(0.305)	0.959(0.850-1.099)	0.120-12.379	0.531-2.036
Duration of T2DM (years)				
<0.5	1.010(0.303)	0.963(0.849-1.107)	0.104-14.104	0.546-2.053
≥0.5	1.006(0.307)	0.961(0.860-1.091)	0.134-40.089	0.555-1.966
CCI at baseline				
0	1.005(0.289)	0.962(0.856-1.093)	0.108-20.452	0.545-1.985
≥1	1.018(0.319)	0.961(0.839-1.119)	0.162-7.870	0.538-2.133
**Cancer of specific site**				
Gastric	1.006(0.287)	0.962(0.857-1.095)	0.112-24.417	0.558-1.979
Colorectal	1.006(0.287)	0.962(0.857-1.095)	0.112-24.417	0.558-1.979
Liver	1.006(0.287)	0.962(0.857-1.095)	0.112-24.417	0.558-1.979

Pancreas	1.006(0.287)	0.962(0.857-1.095)	0.112-24.417	0.558-1.979
Lung	1.006(0.287)	0.962(0.857-1.095)	0.112-24.417	0.558-1.979
Breast	1.010(0.290)	0.963(0.857-1.099)	0.111-9.640	0.545-2.053
Prostate	1.007(0.295)	0.962(0.851-1.102)	0.127-16.015	0.549-1.979
Bladder	1.006(0.287)	0.962(0.857-1.095)	0.112-24.417	0.558-1.979
Thyroid	1.006(0.287)	0.962(0.857-1.095)	0.112-24.417	0.558-1.979
Lymphoma & leukemia	1.006(0.287)	0.962(0.857-1.095)	0.112-24.417	0.558-1.979
All other cancers	1.006(0.287)	0.962(0.857-1.095)	0.112-24.417	0.558-1.979
**Sensitivity analyses**				
Latency periods (months), follow-up from the index date				
0	1.006(0.281)	0.964(0.856-1.099)	0.128-31.366	0.558-1.937
12	1.009(0.295)	0.966(0.861-1.097)	0.094-25.630	0.538-1.994
18	1.009(0.307)	0.967(0.865-1.095)	0.082-33.544	0.528-1.998
24	1.009(0.314)	0.968(0.867-1.092)	0.076-30.193	0.516-2.031
Latency periods (months), follow-up after the latency periods				
6	1.012(0.251)	0.982(0.925-1.058)	0.134-18.985	0.557-1.820
12	1.017(0.280)	0.990(0.942-1.050)	0.057-25.164	0.587-1.734
18	1.018(0.265)	0.992(0.946-1.050)	0.053-34.633	0.579-1.725
24	1.019(0.290)	0.993(0.949-1.049)	0.048-38.877	0.562-1.721
Missing data				
Excluding individuals missing FBG or HbA1c at baseline	1.007(0.281)	0.963(0.856-1.098)	0.109-12.788	0.560-1.981
Diagnosis validation				
Hospitalization within one year of cancer diagnosis	1.000(0.232)	0.962(0.857-1.095)	0.112-17.059	0.559-1.971

Abbreviations: T2DM: type 2 diabetes mellitus; TZD: thiazolidinediones; FBG: fast blood glucose; HbA1c: glycated haemoglobin; OGLD: other glucose-lowering drugs except sulfonylureas and insulins.

References

1. Lin H, Tang X, Shen P, et al. Using big data to improve cardiovascular care and outcomes in China: a protocol for the CHinese Electronic health Records Research in Yinzhou (CHERRY) Study. *BMJ Open.* 2018;8(2):e19698.
